# TRPV4-induced inflammatory response is involved in neuronal death in pilocarpine model of temporal lobe epilepsy in mice

**DOI:** 10.1038/s41419-019-1612-3

**Published:** 2019-05-16

**Authors:** Zhouqing Wang, Li Zhou, Dong An, Weixing Xu, Chunfeng Wu, Sha Sha, Yingchun Li, Yichao Zhu, Aidong Chen, Yimei Du, Lei Chen, Ling Chen

**Affiliations:** 1 Department of Physiology, Nanjing Medical University, Nanjing, China; 2 Department of Neurology, Children’s Hospital of Nanjing Medical University, Nanjing, China; 3 Research Center of Ion Channelopathy, Institute of Cardiology, Union Hospital, Tongji Medical College, Huazhong University of Science and Technology, Wuhan, China; 4 Neuroprotective Drug Discovery Key Laboratory of Nanjing Medical University, Nanjing, China

**Keywords:** Cell death in the nervous system, Inflammasome

## Abstract

Activation of transient receptor potential vanilloid 4 (TRPV4) induces neuronal injury. TRPV4 activation enhances inflammatory response and promotes the proinflammatory cytokine release in various types of tissue and cells. Hyperneuroinflammation contributes to neuronal damage in epilepsy. Herein, we examined the contribution of neuroinflammation to TRPV4-induced neurotoxicity and its involvement in the inflammation and neuronal damage in pilocarpine model of temporal lobe epilepsy in mice. Icv. injection of TRPV4 agonist GSK1016790A (GSK1016790A-injected mice) increased ionized calcium binding adapter molecule-1 (Iba-1) and glial fibrillary acidic protein (GFAP) protein levels and Iba-1-positive (Iba-1^+^) and GFAP-positive (GFAP^+^) cells in hippocampi, which indicated TRPV4-induced microglial cell and astrocyte activation. The protein levels of nucleotide-binding oligomerization domain-like receptor pyrin domain containing 3 (NLRP3) inflammasome components NLRP3, apoptosis-related spotted protein (ASC) and cysteinyl aspartate-specific protease-1 (caspase-1) were increased in GSK1016790A-injected mice, which indicated NLRP3 inflammasome activation. GSK1016790A also increased proinflammatory cytokine IL-1β, TNF-α and IL-6 protein levels, which were blocked by caspase-1 inhibitor Ac-YVAD-cmk. GSK1016790A-induced neuronal death was attenuated by Ac-YVAD-cmk. Icv. injection of TRPV4-specific antagonist HC-067047 markedly increased the number of surviving cells 3 d post status epilepticus in pilocarpine model of temporal lobe epilepsy in mice (pilocarpine-induced status epilepticus, PISE). HC-067047 also markedly blocked the increase in Iba-1 and GFAP protein levels, as well as Iba-1^+^ and GFAP^+^ cells 3 d post-PISE. Finally, the increased protein levels of NLRP3, ASC and caspase-1 as well as IL-1β, TNF-α and IL-6 were markedly blocked by HC-067047. We conclude that TRPV4-induced neuronal death is mediated at least partially by enhancing the neuroinflammatory response, and this action is involved in neuronal injury following status epilepticus.

## Introduction

Transient receptor potential vanilloid 4 (TRPV4), a member of the vanilloid transient receptor potential (TRPV) channel family, is selectively permeable to calcium (Ca^2+^). TRPV4 is sensitive to multiple stimuli, and its activation induces Ca^2+^ influx to increase the intracellular free Ca^2+^ concentration ([Ca^2+^]_i_)^1^. Hyperactivation of TRPV4 occurs in pathological conditions (such as acute respiratory distress syndrome, cerebral ischemia and Alzheimer’s disease) and may result in cytotoxicity^2–4^. However, the detailed mechanisms underlying TRPV4-induced cellular injury have not been fully elucidated. Recent studies have reported the involvement of TRPV4 in the inflammatory response, and activation of TRPV4 can increase the production of proinflammatory cytokines. For instance, activation of TRPV4 by stretching leads to the release of interleukin (IL)-1α, IL-1β, IL-6 and IL-8 in lung epithelial cells^5^. TRPV4 deficiency prevents neutrophil responses to inflammatory stimuli in acute lung injury^6^. Increased TRPV4 expression and activation promote IL-1β and IL-6 gene expression in intervertebral disc cells^7^. TRPV4 is abundant in the central nervous system (CNS), including the hippocampus, cortex, thalamus and cerebellum, and is expressed on neurons and glial cells^8^. In the brain, microglia cells and astrocytes are major innate immunity cells that produce proinflammatory cytokines^9^. Inhibition of TRPV4 exerts a neuroprotective effect on infrasound-induced injury by decreasing glial cell-released proinflammatory cytokines IL-1β and TNF-α^10^. The contribution of the increased inflammatory response to TRPV4-related cytotoxicity has attracted increasing attention from researchers.

Epilepsy is one of the most common neurological disorders and is characterized by unpredictable seizures. Neuronal injury is the major characteristic neuropathologic change in human epilepsies and most rodent epilepsy models^11^. Neuronal damage occurs within several hours after status epilepticus (SE) in some temporal lobe epilepsy (TLE) models and induces functional and structural changes in the neuronal network^12^. Changes in this network are linked to the frequency of spontaneous seizures. Accumulating evidence strongly supports the relevance of neuroinflammation in the pathophysiology of epilepsy, which contributes to neuronal damage^13^. Clinical studies have reported increased levels of proinflammatory cytokines (such as IL-6, TNF-α and IL-1β) in serum or cerebrospinal fluid^14^. The production of proinflammatory cytokines increases in the brain tissues of rodent epilepsy models^13^. The application of a TRPV4 antagonist can block hyperthermia-induced seizures in the larval zebrafish forebrain, indicating a role for TRPV4 in the pathogenesis of epilepsy^15^. The activation of TRPV4 could enhance inflammation and induce cytotoxicity, but whether the above effect is involved in neuronal damage during epilepsy remains unknown.

In the present study, we first examined the contribution of inflammation to neuronal injury upon TRPV4 activation. Next, we explored the role of TRPV4 in the inflammation and neuronal damage post status epilepticus in pilocarpine model of temporal lobe epilepsy in mice (pilocarpine-induced status epilepticus, PISE).

## Materials and methods

### Animals

Eight-week-old male mice (ICR, Oriental Bio Service Inc., Nanjing, China) weighing 25–30 g were used in this study. Animals were housed in the Animal Core Facility of Nanjing Medical University. This study was approved by the Ethics Committee of Nanjing Medical University (No. IACUC1601090), and all animal experiments were performed in accordance with the Guidelines for Laboratory Animal Research set by Nanjing Medical University. Each experimental group contained nine mice.

### Drug treatment

TRPV4 agonist GSK1016790A, TRPV4 specific antagonist HC-067047 and cysteinyl aspartate-specific protease-1 (caspase-1) inhibitor Ac-YVAD-cmk were intracerebroventricularly (icv.) injected according to previous studies^16^. Mice were anesthetized and then placed in a stereotaxic device (Kopf Instruments, Tujunga, CA, USA). A guide cannula of 23-gauge stainless steel tubing was implanted into the right lateral ventricle (0.3 mm posterior, 1.0 mm lateral, and 2.5 mm ventral to bregma) and anchored to the skull with stainless steel screws and dental cement. Drugs were first dissolved in DMSO and then in 0.9% saline to a final volume of 2 µl with a final DMSO concentration < 0.2%. Drugs were injected using a 26-gauge stainless steel needle (Plastics One, Roanoke, VA). GSK1016790A (1 μM/mouse) was injected once daily for 3 consecutive days. Ac-YVAD-cmk (200 ng/mouse) was injected 30 min before GSK1016790A injection and subsequently injected once daily for 3 d. HC-067047 (10 μM/mouse) was injected 1 h after SE was terminated and then injected once daily for 3 d. The doses of the above antagonists or agonists were chosen as previously reported^3,16^.

### PISE preparation

Mice were intraperitoneally injected with pilocarpine (300 mg/kg) to induce SE^17^. Methylscopolamine (1 mg/kg) was injected 20 min before pilocarpine injection to antagonize peripheral muscarinic activity. Seizure severity was rated using the Racine scale as follows: category 1, immobility and facial twitch; category 2, head nodding; category 3, forelimb clonus; category 4, rearing; and category 5, rearing and falling^18^. SE was defined as the onset of category 4–5 seizures and was terminated after 2 h using diazepam (10 mg/kg). If animals did not develop category 4–5 seizures 30 min after pilocarpine injection, they were excluded from subsequent parts of the study. Control mice were injected with the same volume of saline.

### Histological examination

Histological examination was performed 8 h after the last injection of GSK1016790A or HC-067047 or 3 d after the onset of SE. Mice were anesthetized and then transcardially perfused with ice-cold phosphate-buffered saline (PBS), followed by 4% paraformaldehyde. After the brains were removed, they were placed in fixative (4 °C) overnight and processed for paraffin embedding. Coronal sections (5 μm) were cut from the level of the hippocampus for toluidine blue staining. For glial fibrillary acidic protein (GFAP) and ionized calcium binding adapter molecule-1 (Iba-1) staining, the brains were coronally sectioned at 40 μm, and free-floating sections were incubated with primary antibodies against GFAP (Cat: MAB360, 1:1000, Millipore, Billerica, MA, USA) or Iba-1 (Cat: ab5076, 1:1000, Abcam, Cambridge, UK) overnight at 4 °C, followed by biotin-conjugated goat anti-mouse IgG (Cat: ab6788, 1:2000, Abcam, Cambridge, UK) and biotin-conjugated rabbit anti-goat IgG antibody (Cat: ab6740, 1:100, Abcam, Cambridge, UK). The surviving neurons, GFAP-positive (GAFP^+^) and Iba-1-positive (Iba-1^+^) cells were observed using a light microscope (Olympus DP70, Olympus Corporation, Tokyo, Japan) and counted as previously described^3,19^.

### Western blot analysis

Western blot analysis was performed on hippocampal samples obtained 8 h after the last injection of GSK1016790A or HC-067047 or 3 d after the onset of SE. Hippocampal protein concentrations were determined by using a BCA Protein Assay Kit (Pierce, Rochford, IL, USA). Equal amounts of protein were separated by SDS-polyacrylamide gel electrophoresis and transferred to PVDF membranes. After blocking using 5% nonfat milk in Tris-buffered saline (TBS)/Tween-20, membranes were incubated with primary antibodies against nucleotide-binding oligomerization domain-like receptor pyrin domain containing 3 (NLRP3, Cat: bs-6655R, 1:1000, Beijing Biosynthesis Biotechnology Co., LTD, Beijing, China), apoptosis-related spotted protein (ASC, Cat: 10500–1-AP, 1:1000, Proteintech Group Inc., Wuhan, China), caspase-1 (Cat: 22915–1-AP, 1:1000, Proteintech Group Inc., Wuhan, China), GFAP (Cat: MAB360, 1:1000, Millipore, Billerica, MA, USA), Iba-1 (Cat: ab5076, 1:1000, Abcam, Cambridge, MA, USA), IL-1β (Cat: AF5103, 1:1000, Affinity Biosciences, Cincinnati, OH, USA), TNF-α (Cat: ab215188, 1:1000, Abcam, Cambridge, MA, USA), IL-6 (Cat: 17590–1-AP, 1:1000, Proteintech Group Inc., Wuhan, China) and glyceraldehyde-3-phosphate dehydrogenase (GAPDH, Cat: AP0063, Bioworld Technology, Minneapolis, MN, USA) at 4 °C overnight. After washing with TBST, the membranes were incubated with a HRP-labeled secondary antibody, developed using an ECL Detection Kit (Amersham Biosciences, Piscataway, NJ) and analyzed using ImageJ software (NIH). Hippocampal samples collected from the hemispheres of three mice were considered a set for Western blot analysis, and the summarized data represent the average of three experimental sets.

### Chemicals

Pilocarpine was obtained from Cayman Chemical Company, and other chemicals, unless otherwise stated, were obtained from Sigma Chemical Company.

### Data analysis

Data are expressed as the means ± SEM and were analyzed with Stata 7.0 software (STATA Corporation, College Station, Texas, USA). Unpaired t test or ANOVA followed by Bonferroni’s post hoc test was used for statistical analysis, and significance levels were set at *P* < 0.05 and *P* < 0.01. The numbers of surviving cells, GFAP^+^ and Iba-1^+^ cells or protein levels in mice that were injected with GSK1016790A (GSK1016790A-injected mice), HC-067047 or Ac-YVAD-cmk were expressed as the percentages of those values in vehicle-injected mice. Protein levels in Ac-YVAD-cmk-treated GSK1016790A-injected mice were normalized to those in vehicle-treated GSK1016790A-injected mice. The numbers of surviving cells, GFAP^+^ and Iba-1^+^ cells or protein levels in PISE mice were normalized to those in control mice. The numbers of surviving cells, GFAP^+^ and Iba-1^+^ cells or protein levels in vehicle-treated PISE mice and HC-067047-treated PISE mice were normalized to those in vehicle-treated control mice.

## Results

### Effect of TRPV4 agonist on microglial cell and astrocyte activation

Glial cells are the central effectors in inflammatory processes in the CNS through cell activation and inflammatory cytokine release. TRPV4 has been detected in microglia and astrocytes. In the present study, we first explored the effect of TRPV4 agonist treatment on changes in glial cells using Iba-1 as a marker for microglial cell and GFAP for astrocyte.

**Fig. 1:**
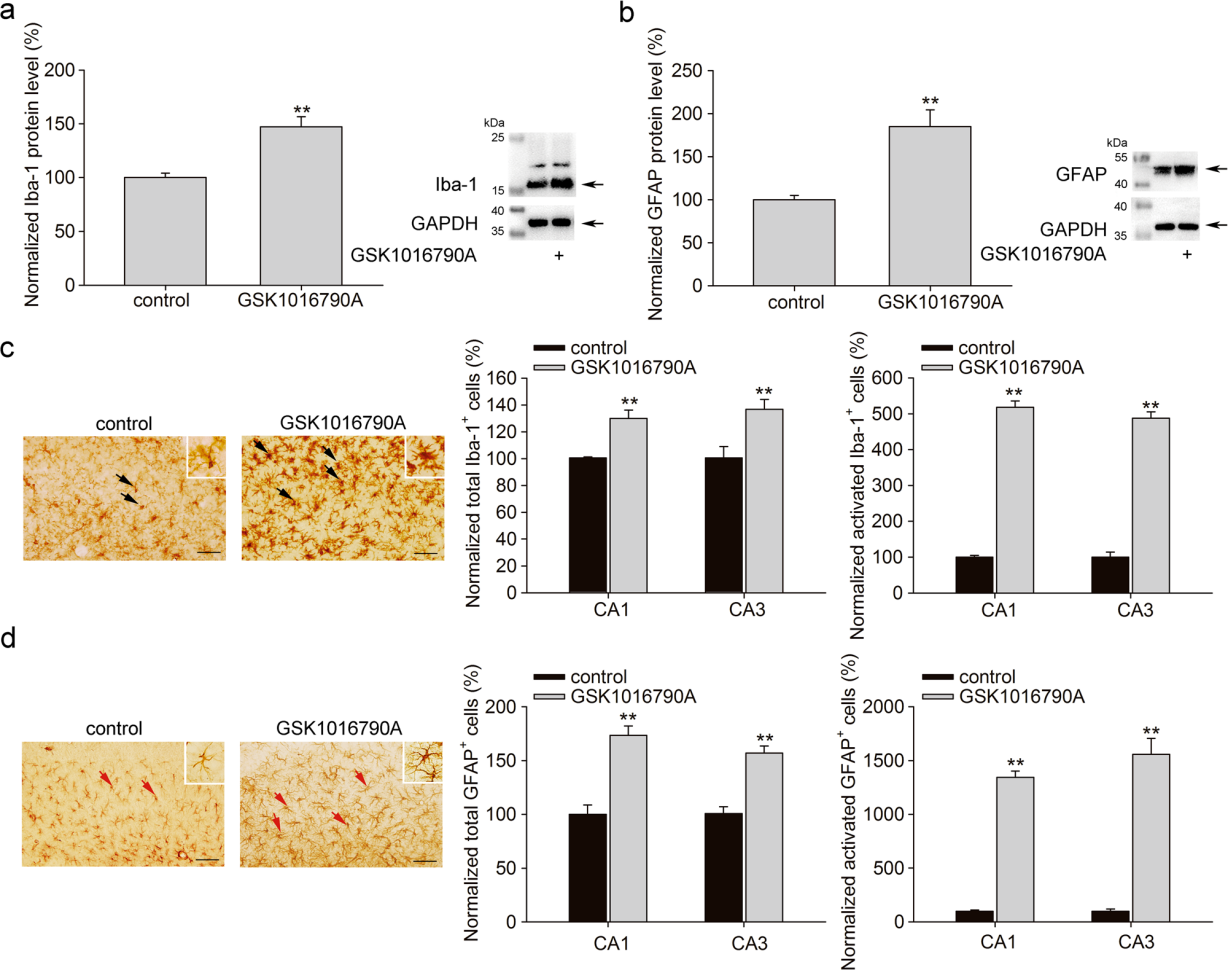
TRPV4 agonist induces microglial cell and astrocyte activation. **a**, **b** Icv. of TRPV4 agonist GSK1016790A markedly increased the protein levels of Iba-1 (**a**) and GFAP (**b**) in hippocampi. **c**, **d** The numbers of total and activated Iba-1^+^ (**c**, black arrow) and GFAP^+^ cells (**d**, red arrow) in hippocampal CA1 and CA3 areas in control and GSK1016790A-injected mice. Iba-1^+^ and GFAP^+^ cells (inset) in GSK1016790A-injected mice reflect the activation state of microglia and astrocyte. Scale bar = 50 μm **P < 0.01 vs. control

Injection of TRPV4 agonist GSK1016790A resulted in significant increases in Iba-1 protein levels in hippocampi (Fig. 1a) and Iba-1^+^ cells in hippocampal CA1 and CA3 areas (Fig. 1c). Moreover, a change in microglial morphology, a hallmark of microglial activation, occurred in GSK1016790A-injected mice. Microglia transitioned from the resting phase to the activated phase, showing amoeboid-like morphology with a large cell body and reduced, retracted processes in response to GSK1016790A treatment.

In this study, we detected significant increases in hippocampal GFAP protein levels and more GFAP^+^ cells in hippocampal CA1 and CA3 areas in GSK1016790A-injected mice (Fig. 1b and d). Activated astrocytes showed morphological changes consistent with hypertrophy with an increased number and length of GFAP-positive processes. GSK1016790A caused a significant increase in activated GFAP^+^ cells (Fig. 1d). These results indicate that activation of TRPV4 results in microglial cell and astrocyte activation.

### Effect of TRPV4 agonist on NRLP3 inflammasome expression and proinflammatory cytokine production

**Fig. 2:**
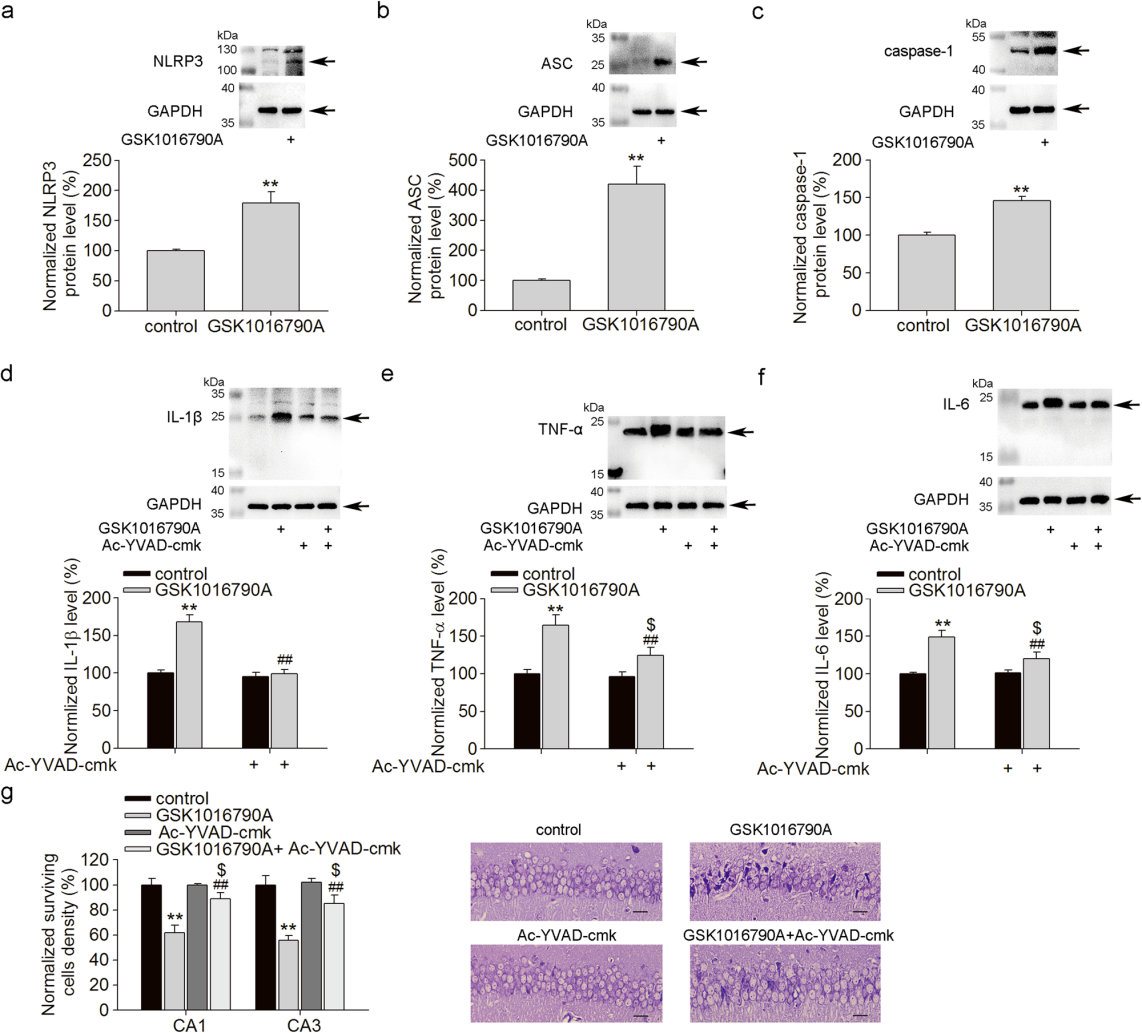
TRPV4 agonist increases NRLP3 inflammasome expression and proinflammatory cytokine production. **a**–**c** The hippocampal protein levels of NLRP3 (**a**), ASC (**b**) and caspase-1 (**c**) in control and GSK1016790A-injected mice. **P < 0.01 vs. control. **d**–**f** Icv. of TRPV4 agonist GSK1016790A markedly increased the protein levels of IL-1β (**d**), TNF-α (**e**) and IL-6 (**f**) in hippocampi. Caspase-1 specific inhibitor Ac-YVAD-cmk completely blocked the increased level of IL-1β and partially attenuated the increased levels of TNF-α and IL-6. **P < 0.01 vs. control, ^##^*P* < 0.01 vs. GSK1016790A, ^$^*P* < 0.05 vs. Ac-YVAD-cmk. **g** Ac-YVAD-cmk markedly attenuated the GSK1016790A-induced decrease in surviving cells in hippocampal CA1 and CA3 areas. Scale bar = 50 μm **P < 0.01 vs. control, ^##^*P* < 0.01 vs. GSK1016790A, ^$^*P* < 0.05 vs. Ac-YVAD-cmk

The NLRP3 inflammasome is composed of several proteins, including NLRP3, ASC and caspase-1, and its activation results in the proteolytic activation of caspase-1 to increase the secretion of proinflammatory cytokine IL-1β^20^. Excessive activation of the NLRP3 inflammasome is involved in neuronal injury in several nervous system diseases^21^. In the present study, the protein levels of NLRP3, ASC and caspase-1 were markedly increased by GSK1016790A treatment (Fig. 2a, b and c). These results indicate that activation of TRPV4 may increase NLRP3 inflammasome activation.

Next, we examined the effect of TRPV4 activation on the production of proinflammatory cytokines and found that the protein levels of IL-1β, TNF-α and IL-6 were higher in GSK1016790A-injected mice than in control mice, indicating an increase in proinflammatory cytokine production (Fig. 2d, e and f). Based on the above results, activation of TRPV4 may enhance neuroinflammation.

Ac-YVAD-cmk is a specific caspase-1 inhibitor^16^. Here, the protein level of IL-1β was markedly lower in mice coinjected with GSK1016790A and Ac-YVAD-cmk than in those injected with GSK1016790A alone (Fig. 2d). Ac-YVAD-cmk showed a lesser effect on the protein level of TNF-α (Fig. 2e) or IL-6 (Fig. 2f) in GSK1016790A-injected mice. TRPV4 activation reportedly induces neuronal injury, and in the present study, more neurons survived in hippocampal CA1 and CA3 areas if GSK1016790A-injected mice were treated with Ac-YVAD-cmk (Fig. 2g). The above results indicate that increased neuroinflammation is involved in TRPV4-induced neurotoxicity.

### Effect of TRPV4 blockade on neuronal death and microglial cell and astrocyte activation post-PISE

**Fig. 3:**
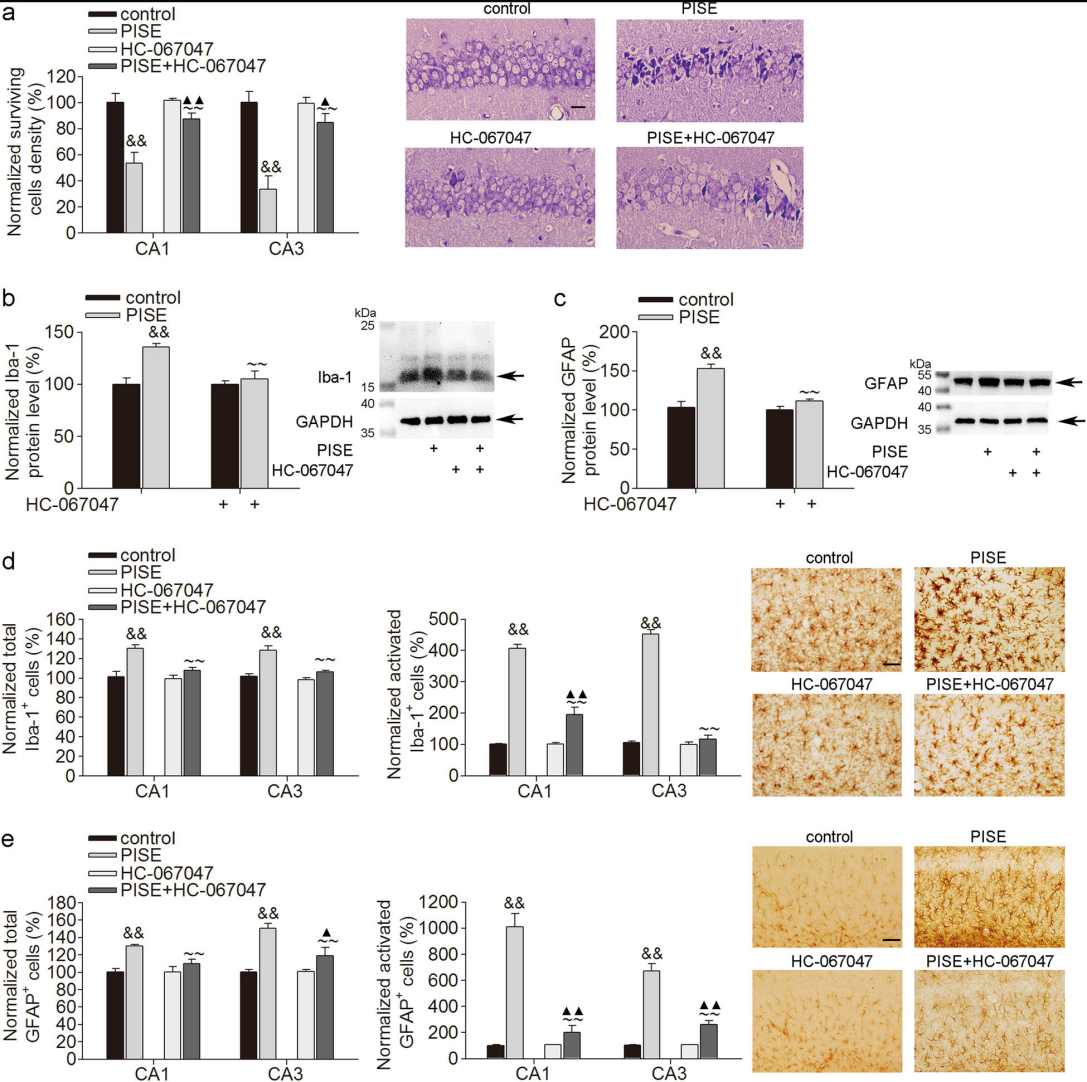
TRPV4 antagonist attenuates neuronal death and activation of microglial cell and astrocyte post-PISE. **a** TRPV4 antagonist HC-067047 markedly increased the number of surviving cells post-PISE. Scale bar = 50 μm ^&&^*P* < 0.01 vs. control, ^~~^*P* < 0.01 vs. PISE, ^$$^*P* < 0.01 vs. control, ^▲^*P* < 0.05, ^▲▲^*P* < 0.01 vs. HC-067047. **b**, **c** HC-067047 decreased Iba-1 (**b**) and GFAP (**c**) protein levels post-PISE. ^&&^*P* < 0.01 vs. control, ^~~^*P* < 0.01 vs. PISE. **d**, **e** HC-067047 attenuated the total and activated numbers of Iba-1^+^ (**d**) and GFAP^+^ cells (**e**) post-PISE. Scale bar = 50 μm ^&&^*P* < 0.01 vs. control, ^~~^*P* < 0.01 vs. PISE, ^▲^*P* < 0.05, ^▲▲^*P* < 0.01 vs. HC-067047

The pilocarpine model of epilepsy is a widely used TLE model, and neuronal cell death has been previously well documented in the PISE model using different mouse strains^22^. In a previous study, pyramidal cell injury in hippocampal CA3 and CA1 was detected as early as 1 d post-PISE and gradually became extensive from 2 to 14 d post-PISE^23^. In the present study, neuronal injury and glial activation in hippocampus were examined 3 d post-PISE. As shown in Fig. 3a, fewer cells survived in hippocampal CA1 and CA3 areas following PISE. In our recent study, TRPV4 protein levels increased from 3 h to 30 d following PISE (unpublished data). In the present study, after administration of TRPV4 specific antagonist HC-067047, more cells survived in hippocampal CA1 and CA3 areas following PISE (Fig. 3a). This result indicates that blocking TRPV4 showed neuroprotection following PISE.

Activation of innate immunity reportedly occurs in different experimental models of epilepsy. In this study, consistent with previous reports, the protein levels of Iba-1 (Fig. 3b) and GFAP (Fig. 3c) were higher, and more Iba-1^+^ (Fig. 3d) and GFAP^+^ (Fig. 3e) cells were found in hippocampal CA3 and CA1 areas in PISE mice than in control mice. Additionally, more activated Iba-1^+^ and GFAP^+^ cells were present in PISE mice. As shown in Fig. 3b-e, the increases in the protein levels of Iba-1 (Fig. 3b) and GFAP (Fig. 3c), as well as in the numbers of total Iba-1^+^ (Fig. 3d) and GFAP^+^ (Fig. 3e) cells, were significantly attenuated by HC-067047 treatment. HC-067047 also decreased the number of activated Iba-1^+^ (Fig. 3d) and GFAP^+^ (Fig. 3e) cells in PISE mice. These results imply that blocking TRPV4 inhibits microglial cell and astrocyte activation following PISE.

### Effect of TRPV4 blockade on the expression of the NRLP3 inflammasome and inflammatory cytokines post-PISE

**Fig. 4:**
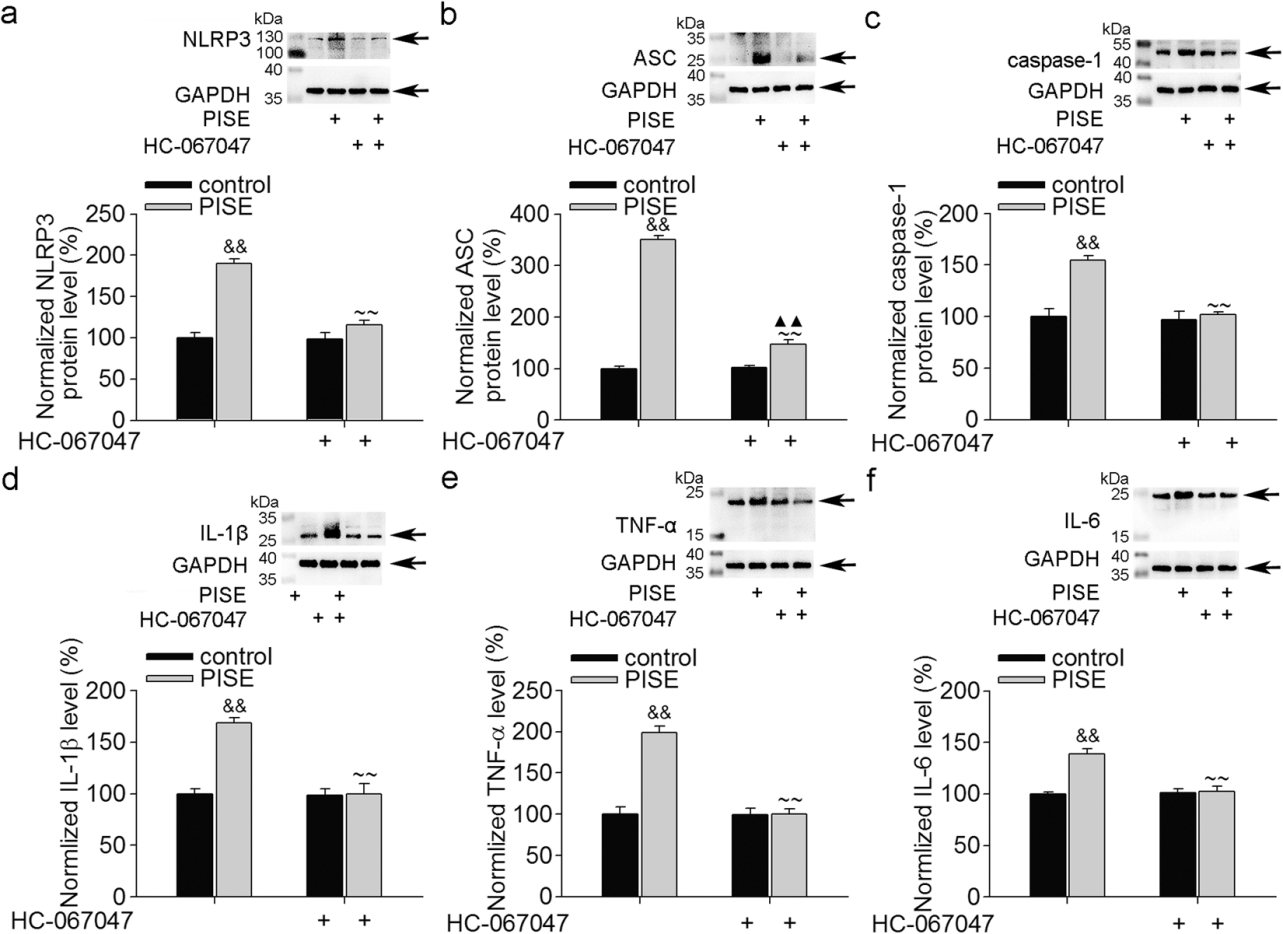
TRPV4 antagonist decreases NLRP3 inflammasome expression and proinflammatory cytokine production post-PISE. **a**–**c** Increased hippocampal protein levels of NLRP3 (**a**), ASC (**b**) and caspase-1 (**c**) post-PISE were markedly attenuated by HC-067047. ^&&^*P* < 0.01 vs. control, ^~~^*P* < 0.01 vs. PISE, ^▲▲^*P* < 0.01 vs. HC-067047. **d**–**f** PISE-induced increases in IL-1β (**d**), TNF-α (**e**) and IL-6 (**f**) protein levels were markedly blocked by HC-067047. ^&&^*P* < 0.01 vs. control, ^~~^*P* < 0.01 vs. PISE

Hyperneuroinflammation has been found in previous TLE models^13^. Activation of the NLRP3 inflammasome contributes to neuronal damage following amygdala kindling-induced SE^24^. As shown in Fig. 4, increases in NLRP3 (Fig. 4a), ASC (Fig. 4b) and caspase-1 (Fig. 4c) protein levels were found following PISE, and these changes were markedly blocked by treatment with HC-067047. Moreover, increases in IL-1β (Fig. 4d), TNF-α (Fig. 4e) and IL-6 (Fig. 4f) protein levels following PISE were significantly attenuated by HC-067047. These results indicate that blocking TRPV4 inhibits neuroinflammation following PISE.

## Discussion

In the CNS, TRPV4 has been detected in neurons as well as astrocytes and microglia^8^. TRPV4 can be activated by a variety of stimuli, including mild temperature changes, hypo-osmolarity-induced mechanical stretch, lipid arachidonic acid, and epoxyeicosatrienoic acid (EET) metabolites. Due to the widespread expression, polymodal activation and Ca^2+^ permeability of TRPV4, it plays an important role in modulating nervous system function. In recent studies, hyperactivation of TRPV4 resulted in neurotoxicity, which is involved in neuronal injury related to intracerebral hemorrhage, traumatic brain injury cerebral ischemia and infrasound-induced neuronal impairment^3,10,25,26^. The mechanisms underlying TRPV4 activation-induced neurotoxicity are complicated and remain to be elucidated. According to previous studies, activation of TRPV4 triggers the endoplasmic reticulum stress through increasing [Ca^2+^]_i_ after intracerebral hemorrhage and blockage of TRPV4 preserves the blood-brain barrier and shows neuroprotection following intracerebral hemorrhage^25,26^. During traumatic brain injury, activation of TRPV4 aggravates the brain edema and neuronal damage through activation of the mitogen-activated protein kinase (MAPK) cascade and protein kinase B (Akt)-related signaling pathway^27^. Activation of TRPV4 may increase glutamate release from the presynaptic membrane and astrocytes, as well as enhance *N*-methyl-d-aspartate (NMDA) glutamate receptor function, which may facilitate glutamate excitotoxicity during the cerebral ischemic injury^28–30^. The application of TRPV4 agonists activate apoptosis-related signaling pathways through inhibiting phosphatidyl inositol 3-kinase (PI3K)/Akt signaling and enhancing p38 MAPK signaling, contributing to the neuronal injury in cerebral ischemia^31^. Activation of TRPV4 may increase the activity and expression of matrix metalloproteinases-9 and thus is involved in the formation of brain edema during cerebral ischemia^32^. Injection of TRPV4 antagonist HC-067047 reduces brain infarction^3^. However, Chen’s research group found that application of TRPV4 agonist may improve the functional recovery from ischemic stroke through increasing angiogenesis and neurogenesis^33^. The different results between Chen’s and others’ reports are likely due to the difference in drug administration. In Chen’s study, TRPV4 agonist 4α-PDD was administered by intravenous injection, while in the studies from other research groups, TRPV4 agonist GSK1016790A or antagonist HC-067047 was administered by icv. injection^3,25,26,28,29,31,32^. It is not clear whether the protective effect of 4α-PDD in the CNS is mediated by 4α-PDD *per se* or by its metabolite that passes through blood-brain barrier, and more experiments are needed to clarify this. Substantial evidence has proven that activation of TRPV4 promotes the release of proinflammatory cytokines and is involved in inflammation in the lung, gastrointestinal system, adipose tissue, retina, etc^34^. In the CNS, neuroinflammation is also an important contributor to neuronal injury in pathological conditions. In the hippocampus, TRPV4 in activated astrocytes is considered to mediate neuronal injury following oxidative stress, infrasound exposure or Aβ treatment^4,10^. TRPV4 activation is responsible for infrasound-induced microglial activation and subsequent neuronal death^10^. TRPV4 inhibition reduces apoptosis of oligodendrocyte induced by LPS-activated microglia and inflammation in a curprizone-induced mouse model of demyelination^35^. However, there is report that LPS-induced microglial activation in the striatum could be inhibited by the TRPV4 agonist 4α-PDD, but this study indicated that the inhibitory effect of 4α-PDD can not be attributed to microglial TRPV4 alone^36^. The divergence in TRPV4 activation during microglial activation may be due to the differences between the brain regions studied and the causes of neuronal injury in different research groups. In the present study, more activated astrocytes and microglia in the hippocampus were found in GSK1016790A-injected mice (Fig. 1), accompanied by increases in NLRP3 inflammasome expression, as well as IL-1β, TNF-α and IL-6 levels (Fig. 2). These findings indicate that activation of TRPV4 may enhance neuroinflammation in the hippocampus. In the present study, GSK1016790A-induced neuronal death was markedly blocked by a caspase-1 inhibitor that can reduce the production of IL-1β (Fig. 2h). This result indicates that enhanced neuroinflammation contributes at least partially to TRPV4 activation-induced neurotoxicity.

The NLRP3 inflammasome is a cytoplasmic complex in which NLRP3 interacts with the adaptor protein ASC to enable the recruitment and activation of caspase-1, leading to the maturation of IL-1β and IL-18^20^. Some factors, such as the elevation of [Ca^2+^]_i_, reactive oxygen species (ROS) production, cytosolic depletion of potassium, and lysosome disruption, are responsible for the activation of this inflammasome^37,38^. In the present study, application of TRPV4 agonist increased the expression of NLRP3, ASC and caspase-1, indicative of the activation of the NLRP3 inflammasome (Fig. 2a-c). Activation of TRPM2, another member of the TRP superfamily that is also permeable to Ca^2+^, resulted in NLRP3 inflammasome activation through mediating Ca^2+^ influx and ROS production^39,40^. Activation of TRPV4 results in the elevation of [Ca^2+^]_i_ by inducing Ca^2+^ flux and enhancing NMDA glutamate receptor-mediated Ca^2+^ influx^8,29^. Enhanced ROS production can also occur following TRPV4 agonist treatment^41^. Activation of TRPV4 increased the tetraethylammonium chloride-sensitive potassium current in trigeminal ganglion neurons, which suggests that activation of TRPV4 may decrease intracellular potassium by enhancing potassium efflux^42^. The specific mechanisms underlying TRPV4-induced NLRP3 inflammasome activation need to be clarified in future work.

TRPV4 activation plays an important role in modulating neuronal excitability and survival. In a study using larval zebrafish, seizure-related neural activity triggered by an increase in brain temperature was blocked by a TRPV4 antagonist^15^. Moreover, TRPV4 expression increased in the cortical lesions of patients with focal cortical dysplasia, a known form of therapy-refractory epilepsy^43^. Our recent unpublished study revealed that TRPV4 protein levels increased 3 h to 30 d post-PISE. These studies imply that TRPV4 may be involved in the pathogenesis of epilepsy. Previous studies have reported subsequent waves of inflammation after the induction of SE, and proinflammatory cytokines are increased in activated astrocytes and microglia in the resected brain tissues of epilepsy patients and model animals^13,14^. Available evidence suggests that seizure-related inflammation might contribute to neuronal injury and synaptic reorganization, which plays an important role during epileptogenesis. Activation of the NLRP3 inflammasome has been detected during epilepsy, and knockdown of NLRP3 or caspase-1 decreased IL-1β and IL-16 levels and provided neuroprotection against SE-associated neuronal damage^13,14^. Consistently, the present study showed neuronal loss and hyperneuroinflammation following PISE (Fig. 3 and Fig. 4). Application of a TRPV4-specific antagonist markedly attenuated neuronal injury following PISE (Fig. 3a). Furthermore, blocking TRPV4 inhibited the activation of microglial cells and astrocytes (Fig. 3), and the increased NLRP3 inflammasome and proinflammatory cytokine production (Fig. 4) post-PISE. Collectively, the present results suggest that TRPV4-mediated inflammation may be involved in neuronal injury following PISE.

**Fig. 5:**
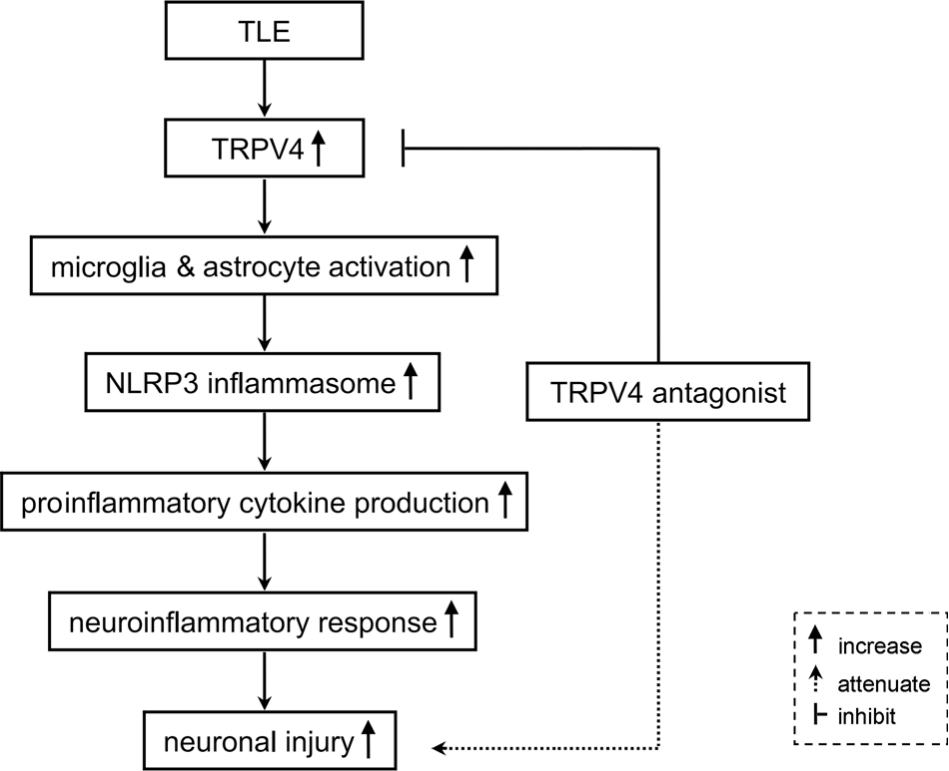
Possible mechanisms underlying the involvement of TRPV4 in neuronal injury in TLE. Activation of TRPV4 leads to microglial cell and astroycte activation, and the increase of NLRP3 inflammasome expression to produce more proinflammatory cytokines, which is involved in the neuronal injury in TLE. Blocking TRPV4 may attenuate neuronal injury post SE through inhibiting immuno-inflammatory response

The present study provided the first evidence that activation of TRPV4 may induce glial cell-mediated neuroinflammation, which contributes to TRPV4-induced neurotoxicity (Fig. 5). Blocking TRPV4 could attenuate neuroinflammation and prevent neuronal injury following PISE. Activation of TRPV4 may facilitate glutamate excitotoxicity, enhance oxidative stress and activate apoptosis-related signaling pathways^3,28,29,31,41^. TRPV4 activation also aid in the destruction of blood-brain barrier and the subsequent brain edema in cerebral ischemia, traumatic brain injury and intracerebral hemorrhage^26,27,32^. Whether the above effects are involved in the neuroprotection of TRPV4 blockade following PISE must be further studied. Pharmacological blockade of TRPV4 function may offer a potential therapy for neuronal injury following epilepsy.
