# Author Correction: TRPV4-induced inflammatory response is involved in neuronal death in pilocarpine model of temporal lobe epilepsy in mice

**DOI:** 10.1038/s41419-019-1691-1

**Published:** 2019-06-21

**Authors:** Zhouqing Wang, Li Zhou, Dong An, Weixing Xu, Chunfeng Wu, Sha Sha, Yingchun Li, Yichao Zhu, Aidong Chen, Yimei Du, Lei Chen, Ling Chen

**Affiliations:** 1 Department of Neurology, Children’s Hospital of Nanjing Medical University, Nanjing, China; 2 Research Center of Ion Channelopathy, Institute of Cardiology, Union Hospital, Tongji Medical College, Huazhong University of Science and Technology, Wuhan, China; 3 Neuroprotective Drug Discovery Key Laboratory of Nanjing Medical University, Nanjing, China; 4 Department of Physiology, Nanjing Medical University, Nanjing, China

**Keywords:** Cell death in the nervous system, Inflammasome

**Correction to: Cell Death and Disease**

10.1038/s41419-019-1612-3 Published online 16 May 2019

The article contains an error in Fig. 3. The correct version of Fig. 3 is shown below. This has now been corrected in both the PDF and HTML versions of the article.

**Fig. 3:**
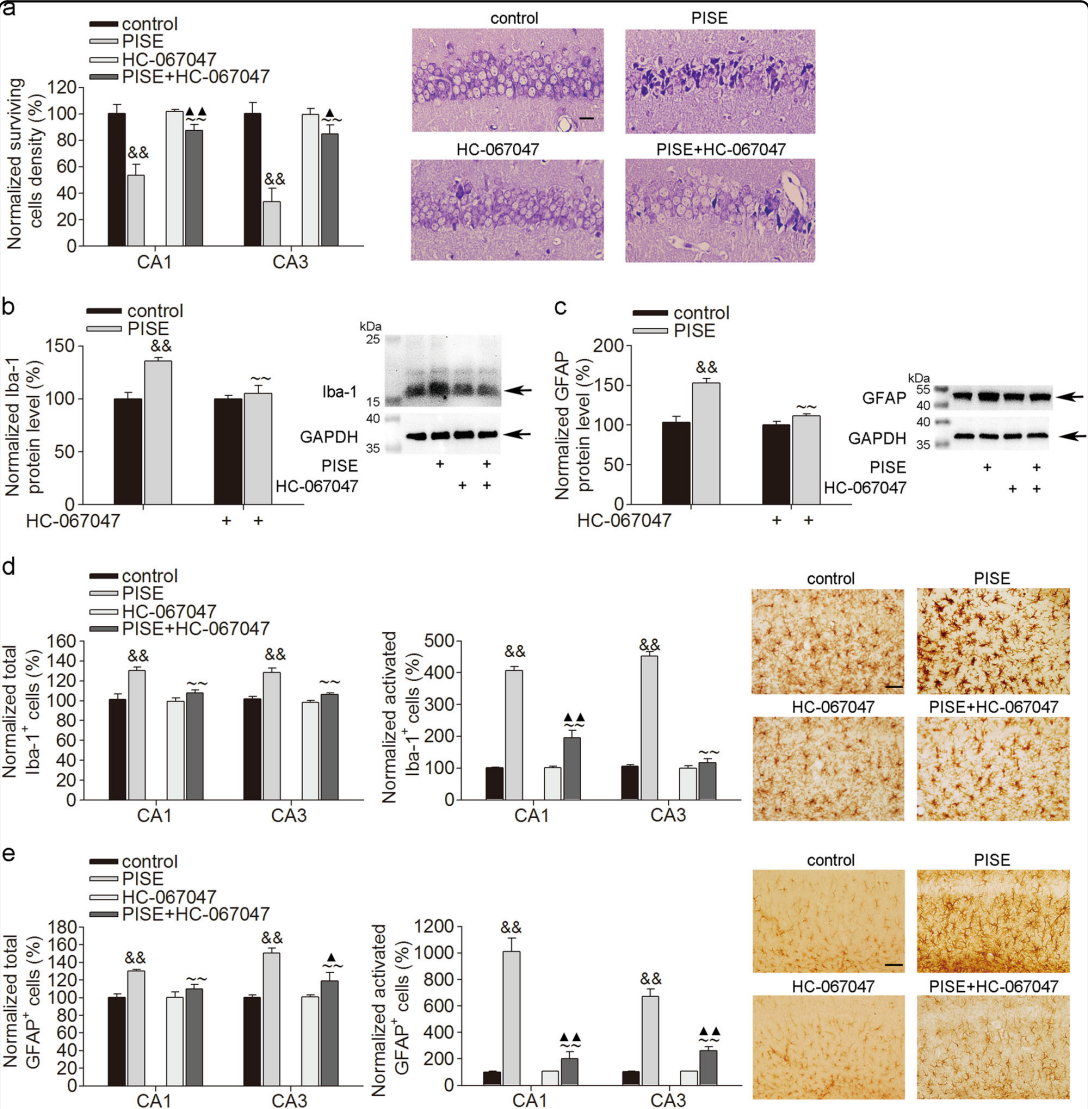
▓

